# Isolation and characterization of bacteriophages for carbapenem resistant *Enterobacter cloacae* complex strains

**DOI:** 10.1038/s41598-025-22703-x

**Published:** 2025-11-06

**Authors:** Matthew Imanaka, Wakana Yamashita, Shinjiro Ojima, Aa Haeruman Azam, Michiyo Kataoka, Tadaki Suzuki, Yo Sugawara, Motoyuki Sugai, Yoshimasa Takahashi, Koichi Watashi, Satoshi Tsuneda, Kotaro Kiga

**Affiliations:** 1 Research Center for Drug and Vaccine Development, National Institute of Infectious Diseases, Tokyo, 162–8640 Japan; 2 Department of Life Science and Medical Bioscience, Waseda University, 2–2 Wakamatsu-cho, Shinjuku-ku, 162–8480 Tokyo Japan; 3 Department of Infectious Disease Pathology, National Institute of Infectious Diseases, Japan Institute for Health Security, Tokyo, 162–8640 Japan; 4 Department of Infectious Disease Pathobiology, Graduate school of Medicine, Chiba University, Chiba, 260-8670 Japan; 5 Antimicrobial Resistance Research Center, National Institute of Infectious Diseases, Tokyo, Japan; 6 Phage Therapy Institute, Comprehensive Research Organization, Waseda University, 2–2 Wakamatsu-cho, Shinjuku-ku, 162–8480 Tokyo Japan

**Keywords:** Bacteriophages, Antimicrobial resistance

## Abstract

There has been an increased interest in phage therapy for the treatment of antimicrobial resistant bacterial infections due to the decreasing effectiveness of antibiotics. While phage therapy has been successful, it often requires time to isolate and characterize phages suitable for phage therapy. Here, we isolated and characterized three phages against carbapenem-resistant *Enterobacter cloacae* complex (ECC) strains to potentially be applied in phage therapy. ECC strains commonly cause sepsis and urinary tract infections in hospitals and are designated as a pathogen of critical importance for its global prevalence and high instance of antimicrobial resistance. Initially, a total of 96 phages were isolated using a collection of diverse clinical ECC strains. Three phages were selected for genome sequencing and additional characterization based on their broad host range and lytic activity. Based on average nucleotide identity (ANI), vB_ECC_YI837 and vB_ECC_MY742 were considered strains of previously isolated phages vB_CsaM_leN and Entb_45, respectively. One phage had an ANI of 92.65% and was therefore considered novel and named vB_ECC_CW742. Furthermore, none of the phages carried toxin, antimicrobial resistance, or lysogeny-related genes. Collectively, the phages covered 82% (108/131) of our carbapenem-resistant ECC strain library. These findings suggest that the three phages are promising candidates for phage therapy against carbapenem-resistant ECC infections.

**Supplementary Information:**

The online version contains supplementary material available at 10.1038/s41598-025-22703-x.

## Introduction

The spread of antimicrobial resistance (AMR) is a rapidly developing threat that traditional antimicrobials struggle to address. As such, there has been a growing interest in alternative approaches such as phage therapy. Phage therapy utilizes bacteriophages (phages) to selectively infect and kill pathogenic bacteria. Whereas traditional antimicrobials completely destroy the microbiota possibly leading to dysbiosis, phage therapy selectively targets the pathogen while preserving the microbiota composition. This high specificity coupled with the easily producible nature of phages makes phage therapy a promising therapeutic option. While bacterial hosts can become resistant to phage infection, the diversity of phages enables the isolation of new phages targeting phage-resistant strains. Phage therapy has been implemented successfully but treatments are often highly specific against the target pathogenic strain and cannot be widely utilized. Phages recognize their hosts by structures on the host cell wall such as pili, membrane transporters, and other structural elements^1^. Even specific sugar residues are often required for host recognition^2^. Furthermore, as phages and their bacterial hosts have undergone billions of years of coevolution, bacteria have evolved a variety of phage-defense system to protect themselves from phage infection after receptor recognition^3,4^. The highly specific nature of phages often requires the isolation and characterization of phages on a case-by-case basis. As such, a phage with a broad host range and strong lytic activity is an ideal option for phage therapy applications. Here, we describe the isolation and characterization of phages effective against carbapenem-resistant *Enterobacter cloacae* complex (ECC) strains for the potential future use in phage therapy.

ECC is a group of *Enterobacter* species with high genome similarities: *E. cloacae*,* E asburiae*,* E. hormaechei*,* E. kobei*,* and E. ludwigii*^5^. ECC strains are nosocomial pathogens that cause pneumonia, sepsis, and, urinary tract infections in intensive care and neonatal intensive care units^6^. Paired with the high levels of antibiotic resistance, medium-low treatability, and growing prevalence, carbapenem-resistant *Enterobacter* species are classified as an ESKAPEE pathogen of critical importance^7^. While Japan has a relatively low instance of carbapenem-resistant ECC infections at 1–2%, the carbapenemase genes can vary greatly, with bla_IMP−1_ being detected 62% of the time in resistant strains^8^. As a diverse array of carbapenem-resistant genes can be easily spread by horizontal gene transfer, there is the possibility of future outbreaks, and thus, an incentive to develop phage therapeutics. ECC outbreaks are also often country specific with certain sequence types (STs) being more prevalent than others. For example, while ST 78 is commonly isolated from hospitals in Japan^8^, ST 114 is more widespread in Australia^9^. Considering these factors, we aimed to isolate lytic phages with broad host ranges for potential use in future phage therapy applications in Japan.

## Results

### Host range

**Fig. 1:**
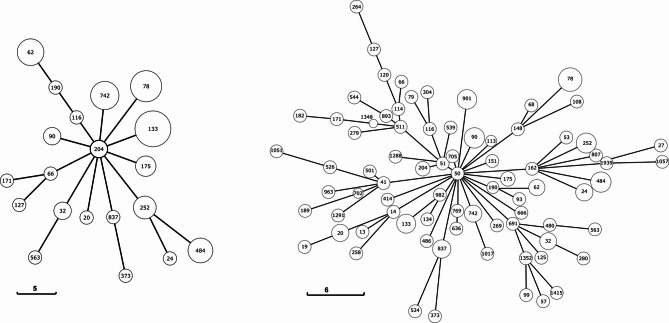
Minimum spanning tree based on MLST. The size of each node represents the relative number of strains for each ST and lines represent the evolutionary distance based on MLST. Our initial collection contained 20 unique STs across 63 strains (left). An additional 68 strains covering 59 additional STs (right) were added to bolster analysis.

Initially, we received 63 ECC strains isolated at hospitals across Japan (Supplementary Table 1). The 63 ECC strains contained 20 unique STs. An initial selection of 8 strains (ST 20, 66, 133, 190, 204, 252, 742, 837) were chosen based on the minimum spanning tree (MST) created from the multi-locus sequence typing (MLST) (Fig. 1).

**Fig. 2:**
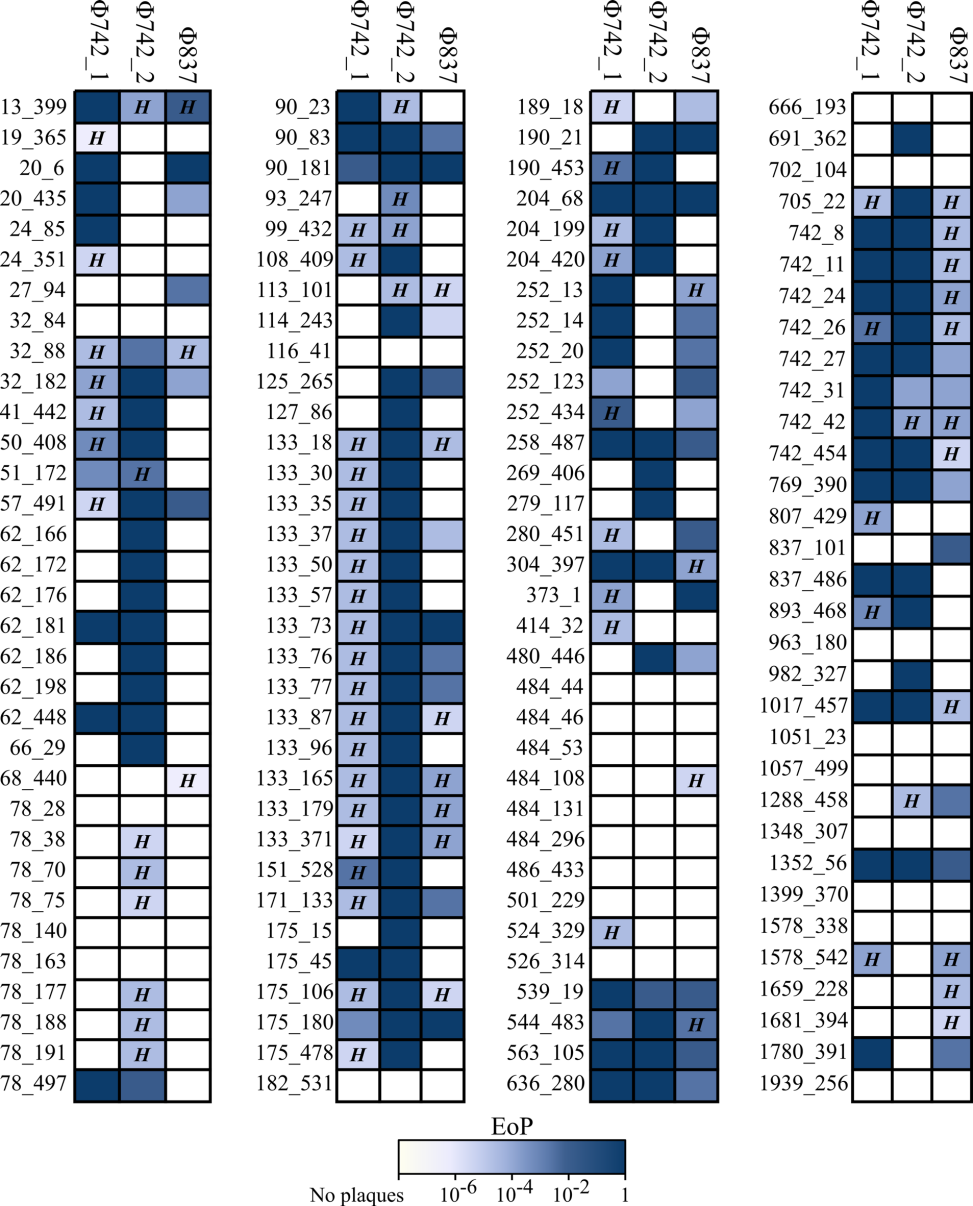
Host range of Φ742_1, Φ742_2, and Φ837. The numbers beside each tile indicate the bacterial host ST followed by the catalog entry within our library (13_399: ST 13, #399). Hazy plaques are notated by *H.* Efficiency of plating (EoP) was calculated from the titer of the phage against the selected host divided by the titer when plated against the isolation host. Φ742_1, Φ742_2, and Φ837 are referred to as vB_ECC_CW742, vB_ECC_MY742, and vB_ECC_YI837 following genome sequencing.

This selection was made considering the number of isolates of an ST and the relative relatedness to other STs. Phages were isolated from purified sewage from Tokyo, Japan. A total of 96 phages, 12 plaques for each bacterial strain, were isolated, purified, propagated, and screened by spot testing against the other target strains (Supplementary Fig. 1). Three phages, Φ742_1, Φ742_2, and Φ837, were selected for further study. Φ742_2 was selected for further study as it was strongly lytic against five of the eight isolation hosts (Supplementary Fig. 1). Φ742_1 was selected to target two strains uncovered by Φ742_2. Φ837 was selected as it was one of the few phages capable of infecting ST 837 while maintaining moderate infectivity against the other strains. Phages Φ742_1 and Φ742_2 were isolated using ST 742 (*Enterobacter hormaechei*, ST 742, #27) as a host, and Φ837 was isolated using ST 837 (*Enterobacter cloacae*, ST 837, #101). After receiving an additional 68 ECC strains (Supplementary Table 1), the three phages were spot tested against the full carbapenem-resistant ECC library. Φ837 covered 47% (61/131) of the ECC library, Φ742_1 covered 59% (77/131), and Φ742_2 covered 64% (84/131) (Fig. 2).

Despite being isolated using the same host, Φ742_1 and Φ742_2 showed distinct host ranges and lytic activity. Φ742_1 covered 18 strains uncovered by Φ742_2, and Φ742_2 covered 25 strains uncovered by Φ742_1. Of the three phages, Φ742_2 possessed the strongest infectivity as it maintained the highest EoP across the most clinical isolates.

Of the 23 ECC strains not covered, six belonged to ST 484 and three belonged to ST 78. Additionally, while six of the ten ST 78 strains were targeted by Φ742_2, there was a much lower efficiency of plating (EoP) and produced hazy plaques (Fig. 2). After these findings, phages against ST 78 and ST 484 were also isolated and their host range was checked by spot testing (Supplementary Fig. 2). Φ78_1 covered the ST 78 strains and had a relatively broad host range of 51% (66/131) of the collection. Conversely, Φ484_1 only targeted ST 484 strains, covering only 9% (12/131) of the collection. Further genomic analysis and characterization is needed to verify the suitability of Φ78_1 and Φ484_1 for therapeutic uses.

### Genome analysis

**Fig. 3:**
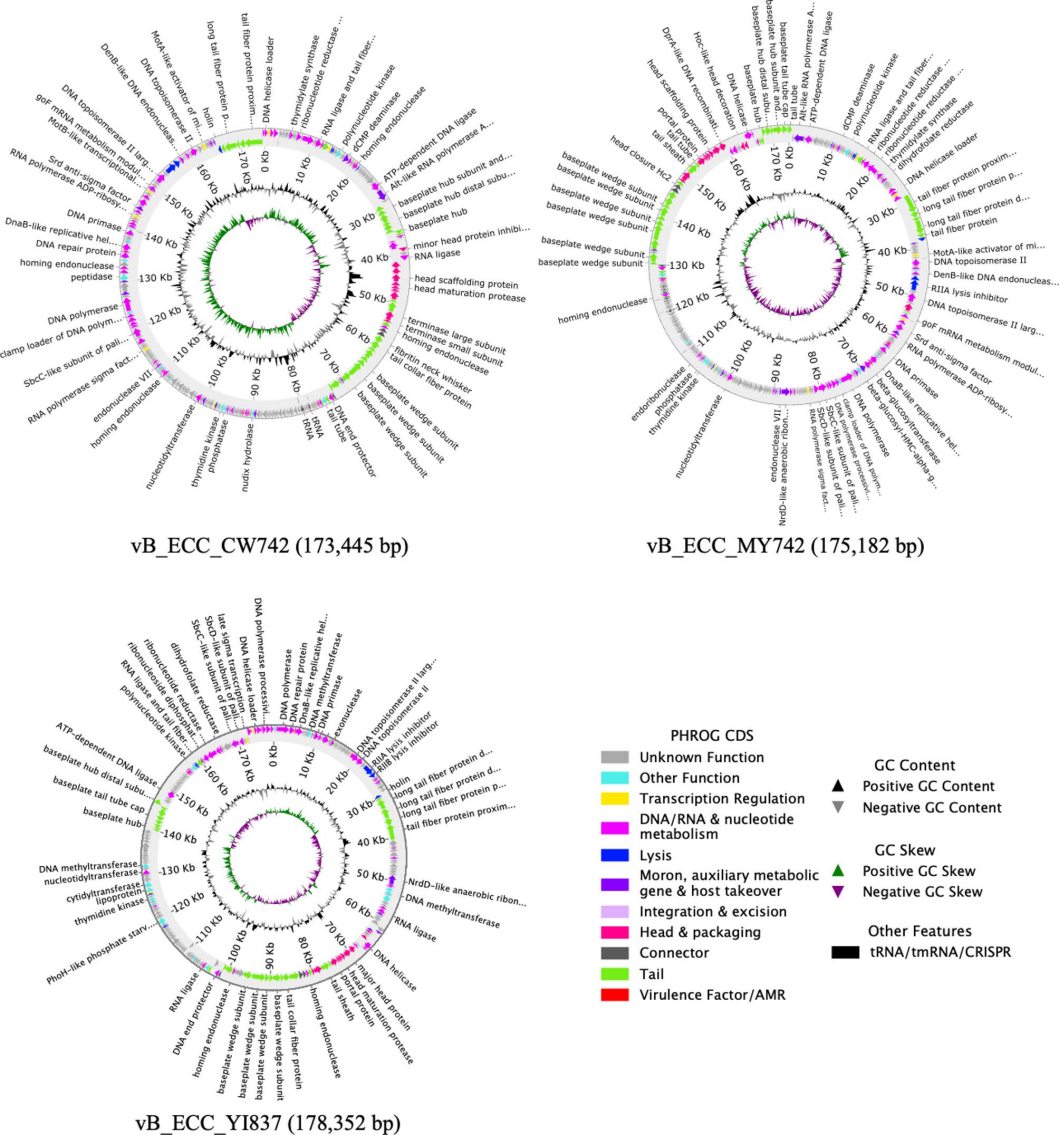
Genome arrangement of the three isolated phages. Genome maps were visualized using Pharokka.

The phage genomes were then sequenced to determine their suitability for phage therapy applications. The genome size of Φ742_1 was found to be 173,445 bp and was registered to GenBank under PV019367 and named vB_ECC_CW742. vB_ECC_CW742 had a 92.65% average nucleotide identity (ANI) and a VIRDIC similarity of 87.98% to the closest related phage, *Enterobacter* phage vB_EluP_RZH (PQ140450) (Fig. 3, Supplemental Supplementary Table 2).

Φ742_2 possessed a genome size of 175,182 bp and named vB_ECC_MY742 under GenBank accession PV593737. vB_ECC_MY742 had a 95.99% ANI identity to *Enterobacter* phage Entb_45 (ON630910.1) and VIRDIC similarity of 91.16% to Enterobacter phage vB_EclM_CIP9 (NC_048849.1) (Supplementary Table 3). Φ837 possessed a genome size of 178,352 bp and named vB_ECC_YI837 under GenBank accession PV593738. vB_ECC_YI837 had a 96.45% ANI identity to *Cronobacter* phage vB_CsaM_leN (KX431560) and a VIRDIC similarity of 96.24% to Cronobacter phage D6b (PV417214.1) (Supplementary Table 4). Following the ICTV guideline considering ANI < 95% as novel, vB_ECC_MY742 and vB_ECC_YI837 were considered closely related strains of previously isolated phages and vB_ECC_CW742 was considered a novel phage^10^. All phages carried no virulence factors, antimicrobial resistance genes, or integration and excision genes, confirming each as possible candidates for phage therapy applications (Supplementary Table 5).

**Fig. 4:**
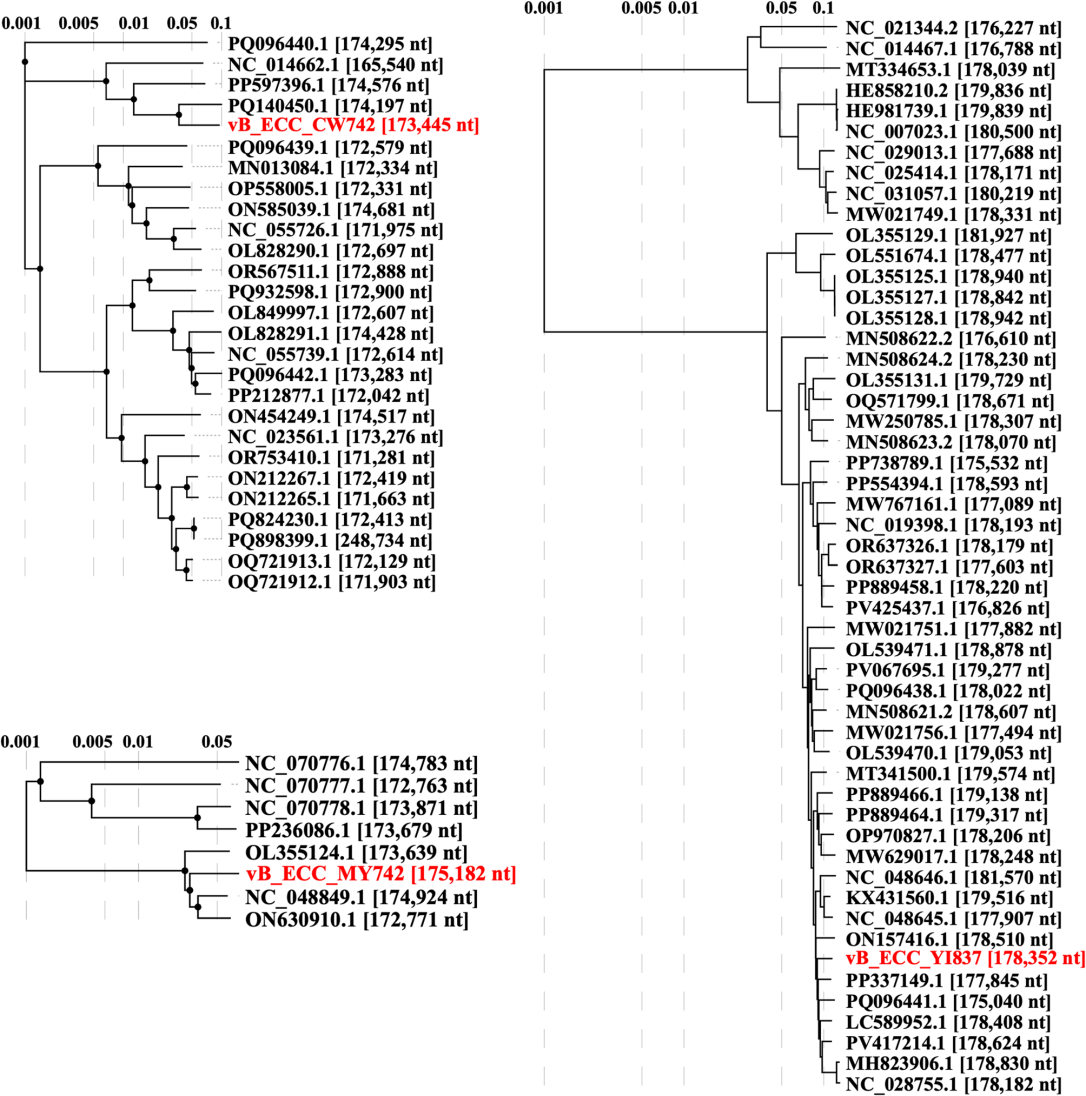
Proteomic tree of vB_ECC_CW742, vB_ECC_MY742, and vB_ECC_YI837. Phages were compared to the BLAST results of the top hits (Query coverage ≥ 80%, Percent Identity ≥ 80%). ViPTree web-service (https://www.genome.jp/viptree/) was used to construct the figure.

Next, phylogenetic analysis found all phages are grouped with other phages of the *Straboviridae* family of bacteriophages, with vB_ECC_CW742 and vB_ECC_MY742 falling in the *Karamvirus* genus and vB_ECC_YI837 falling in the *Pseudotevenvirus* genus (Fig. 4).

Despite the same isolation host and relative relatedness, vB_ECC_CW742 and vB_ECC_MY742 shared an ANI of only 68.83% and VIRIDC similarity of 38.22. vB_ECC_CW742 and vB_ECC_MY742 were grouped with other *Enterobacter* phages, while vB_ECC_YI837 was grouped with *Citrobacter*, *Escherichia*, *Cronobacter*, *Klebsiella*, and *Enterobacter* phages (Fig. 4, Supplementary Fig. 3). vB_ECC_CW742 and vB_ECC_MY742 seem to come from more unique evolutionary lineages as shown by the relative distance to other registered phages. vB_ECC_YI837 is more closely related to a variety of phages. These results also align closely to the VIRDIC scores (Supplementary Tables 2, 3, 4).

### Phage morphology

**Fig. 5:**
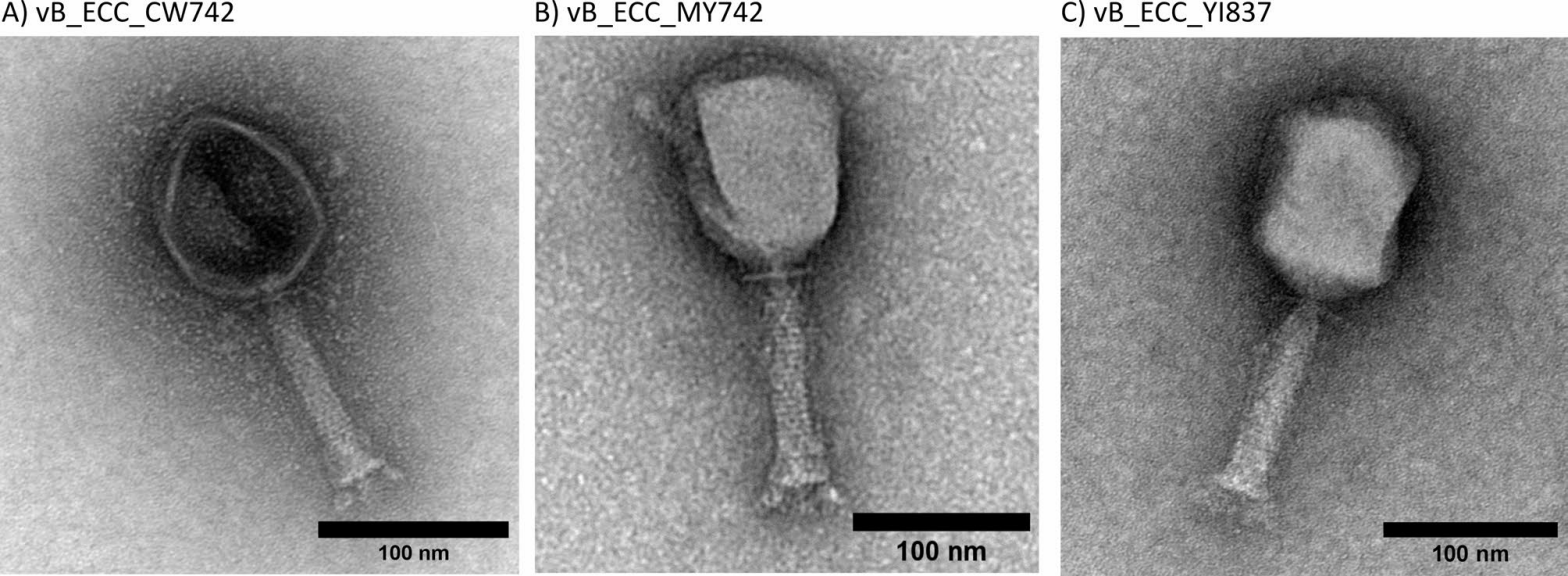
Electron micrograph of the (**A)** vB_ECC_CW742, (**B)** vB_ECC_MY742 and (**C)** vB_ECC_YI837. The scale bars represent 100 nm.

The three phages were imaged by transmission electron microscope (TEM) to determine their morphology. For each phage, the average and standard deviation was taken of three representative viral particles. All three phages showed traits common to myophages, often defined by the relatively large head and contractile tails (Fig. 5).

vB_ECC_CW742 had a head length of 103 ± 0.5 nm and width of 81 ± 2.7 nm, with a tail length of 104 ± 0 nm and width of 19 ± 1 nm. vB_ECC_MY742 had a head length of 123 ± 8 nm and width of 91 ± 0.5 nm, with a tail measuring 110 ± 0.5 nm by 24 ± 0.5 nm. vB_ECC_YI837 had similar measurements, with a head length of 119 ± 0.5 nm and width of 86 ± 2.7 nm, with a tail length of 113 ± 2.5 nm and width of 22 ± 0.2 nm.

### One-step growth

**Fig. 6:**
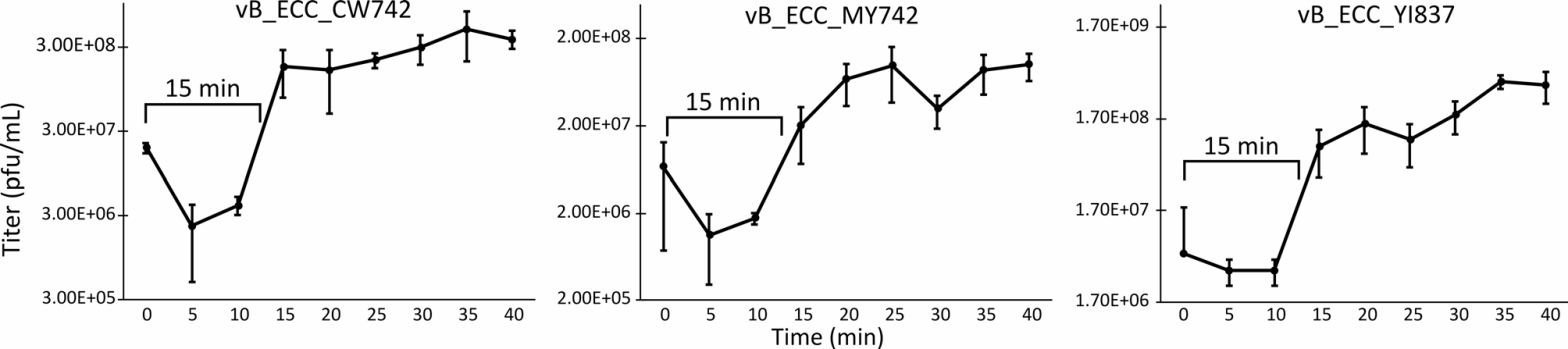
One-step growth curves of three isolated phages. Samples were taken every 5 min for 40 min. The latent period was defined as the period before the sharp increase in phage titer. The average of four replicates was plotted with the error bars representing the standard deviation.

The growth characteristics of the three phages were evaluated by one-step growth curve. The isolation host for each phage was used to construct the corresponding growth curves (Fig. 6).

From the one-step growth curves, all phages had a latent period of around 15 min. vB_ECC_CW742 had a burst size of 19 viral particles per infected cell, vB_ECC_MY742 of 14 particles per cell, and vB_ECC_YI837 of 69 particles per cell. Additionally, all phages suppressed growth of their respective hosts at MOI’s as low as 0.1 (Supplementary Fig. 4).

## Discussion

Here, we isolated three phages, two closely related subspecies and one novel, and evaluated their suitability for phage therapy against carbapenem-resistant ECC strains. The carbapenem-resistant clinical isolates used in this study were collected from hospitals across Japan from the Japan Antimicrobial Resistant Bacterial Surveillance on Gram-negative Rods (JARBS-GNR)^8^. Initially, we isolated 96 phages from sewage taken from Tokyo, Japan using eight ECC clinical strains. These initial three phages were chosen as they possessed unique host ranges against the initial screening set and were strongly lytic. Two phages, vB_ECC_MY742 and vB_ECC_YI837, were found to be closely related to previously isolated phages while vB_ECC_CW742 was found to be a novel phage. vB_ECC_MY742 covered 64% (84/131) of our carbapenem-resistant ECC library, vB_ECC_YI837 covered 47% (61/131), and the novel *Enterobacter* phage vB_ECC_CW742 covered 59% (77/131). Similar studies isolating phages against ECC strains have found host ranges anywhere from 10 to 50% of their ECC library^11,12^. As our phages have host ranges from 47 to 64%, the phages isolated here perform similarly or slightly outperform these previous reports. As our collection is made up of clinical strains, with the three most abundant strains being ST 72, 133, and 742, the broad host ranges could be the result of our phages being specialized against clinical strains. For example, the isolated phages could target membrane features that confer a fitness advantage in the clinic. However, more work is needed to verify this hypothesis.

To our knowledge, this work is one of the few studies that isolated ECC-specific phages based on ST. From this, we gained the novel insight that ECC ST 78 and ST 484 are highly resistant to phage infection. This is particularly important as ST 78 has been reported to be one of the most prevalent strains isolated in hospitals in Japan^8,13^. We initially selected bacterial hosts for phage isolation based on the genetic diversity of our ECC collection. However, because ST 78 is a prevalent strain and is resistant to phage infection, there is incentive to isolate phages effective at overcoming the resistance mechanisms of ST 78 strains. The increased resistance to phage infection could be a result of a phage defense system unique to ST 78 as suggested by the lower EoP and hazy plaques when infected by vB_ECC_MY742. Annotation by DefenseFinder found the Olokun and RADAR II defense systems unique to ST 78 strains^14–16^. While RADAR has been relatively studied^17^, Olokun has not be thoroughly characterized^18^. It may be the case that Olokun confers broad defense against phages but requires experimental validation. Future work is planned to further investigate ST 78 and prepare a cocktail specifically against ST 78 strains.

Genome analysis found that the three characterized phages carried no virulence genes, antibiotic resistance genes, or lysogeny-related gene, making them viable candidates for phage therapy applications. This is an important consideration in phage therapy as unwanted horizontal gene transfer of these elements may exacerbate the patient’s condition. Compared to the other phages isolated in this study, vB_ECC_MY742 had the broadest host range and was the strongest lytic phage. vB_ECC_MY742 also carried slightly more auxiliary metabolic genes and host takeover genes (Supplementary Table 5). As over half of ORFs of vB_ECC_MY742 have unknown functions, there are likely additional auxiliary genes and host takeover genes that enables its unique physiology, albeit not enough to strongly infect ST 78. Additionally, vB_ECC_CW742 carried 19 tRNAs, significantly more than nine annotated in vB_ECC_MY742 and one in vB_ECC_YI837. The various tRNAs may enable vB_ECC_CW742 to overcome translational bottlenecks or tRNA-degrading defense systems. vB_ECC_CW742 and vB_ECC_MY742 also carry adenosine diphosphate (ADP)-ribosyltransferase (ART), which is known to use NAD as a substrate to disrupt host translation machinery^19^. As this is unique to these two phages, its inclusion could be a contributing factor to the broader host range compared to vB_ECC_YI837. Additionally, the relationship between phages vB_ECC_CW742 and vB_ECC_MY742 to ST 133 strains can possibly lead to the identification of new phage-defense system interactions. vB_ECC_CW742 produces hazy plaques on ST 133 strains while vB_ECC_MY742 can strongly infect ST 133 strains (Fig. 2). The hazy plaques produced by vB_ECC_CW742 suggest that the host defense systems are inhibiting strong infection, while vB_ECC_MY742 is unaffected. Differences between the two phages could elucidate the functions of the defense systems of ST 133 and warrant future investigation.

All phages carry similar lytic genes, including a holin, RIIA/RIIB lysis inhibitors, two Rz-like spanin genes, and an endolysin. Both vB_ECC_CW742 and vB_ECC_MY742 carry endolysins similar to T4 lysozyme according to HHpred^20^. In contrast, vB_ECC_YI837 has an endolysin with homology to L-alanyl-D-glutamate peptidase. Despite the different endolysin genes, all phages had similar plaque morphology. Additionally, even though all phages carried similar lysis inhibition systems, vB_ECC_YI837 had a much higher burst size of 69 particles per cell compared to 19 virions per cell of vB_ECC_CW742 and 14 virions per cell of vB_ECC_MY742. As the three phages have similar morphologies, genome sizes, and lytic genes, the increased burst size is likely due to host-specific interactions. Furthermore, as vB_ECC_YI837 has the smallest host range, there may be an evolutionary tradeoff in host range and burst size. In addition to the lytic enzymes, the tail fiber proteins also differed. A S74 peptidase domain was found in the distal long tail fiber of vB_ECC_CW742, possibly accounting for its broad host range compared to other reported *Enterobacter* phages. Future work is planned to generate phage-resistant mutants to identify the specific receptors needed for successful phage infection.

While the phages in this study show strong therapeutic potential in vitro, there are limitations to their viability in the clinic. The phages collectively covered 82% (108/131) of the collection, however, the host range was determined individually by spot test and may change when used in a cocktail. When creating a phage cocktail, it is important to utilize physiologically unique phages to ensure bacterial hosts cannot adapt resistances to multiple phages through a single mutation. Here, phage-resistant mutants appeared around six hours following the initial infection by phage (Supplementary Fig. 4). Future work is planned to identify the mechanisms that confer resistance to the three phages to ensure a single mutation does not confer resistance to all three phages. Additionally, the receptors targeted by each phage should be identified to ensure each phage targets a unique receptor. If multiple phages within a cocktail target the same receptor, the cocktail can become ineffective if the host mutates that specific receptor. As such, before a cocktail is attempted, the receptors for ECC_MY742, vB_ECC_CW742, and vB_ECC_YI837 should be identified to ensure there is no overlap between the phages. While previous studies have indicated that diversity in the LPS O-antigen can influence phage adsorption in Enterobacteriaceae, our genomic comparisons did not identify any ECC-specific LPS features that could account for the host range observed in our phages^1^. Another area to improve is the burst size of vB_ECC_MY742 and vB_ECC_CW742. Both vB_ECC_MY742 and vB_ECC_CW742 had relatively low burst sizes with 14 and 19 viral particles per cell, respectively. To address the issue of low burst size, one possible approach is employ utilize phage training^21,22^. Phage training, or the process of coevolving phages with their hosts, can be used to increase the lytic capacity of phages by increasing burst size or shortening latent period. More work is needed to determine the actual performance of these three phages within a cocktail in liquid assays.

In this study, we used clinical ECC isolates to isolate phages with the goal of identifying phages suitable for therapeutic applications. Using the clinical isolates, we successfully isolated phages that have strong lytic activity, broad host ranges, and lack of virulence factors or antimicrobial resistance genes. Our results here suggest these phages are promising candidates for phage therapy applications. Next, the receptors of each phage should be identified to ensure the phages do not share a common receptor. Additionally, the relatively low burst size of vB_ECC_MY742 and vB_ECC_CW742 could be improved by phage training. Overall, the phages here represent a key start to formulating a cocktail against carbapenem-resistant ECC strains common to Japan and warrant further cocktail and in vivo validation.

## Materials and methods

### Bacterial strains

The carbapenem-resistant clinical isolates used in this study were collected from hospitals across Japan through the Japan Antimicrobial Resistant Bacterial Surveillance on Gram-negative Rods (JARBS-GNR)^8^. Some isolates are available from the Japan Antimicrobial Resistant Bacterial Bank as IMP-type carbapenemase or Carbapenemase panels (https://jarbb.jp/en/). Specimen collection was conducted with informed consent from each participant and was approved by the Institutional Review Board (IRB) of the National Institute of Infectious Diseases (Approval Np. 1251). The initial collection contained 63 different strains across 20 sequence types (STs). GrapeTree v.1.5.0 running MSTTreeV2 was used to create a minimum spanning tree (MST) based on multi-locus sequence typing (MLST)^23^. The tree was used to best select isolation strains to best cover the tree considering relatedness and number of isolates. An additional 68 ECC isolates were added to our library to bolster analysis. An MST containing the additional isolates was constructed as previously mentioned. The information of the isolation location, BioSample accession, and ST can be found in Supplementary Table 1. All experiments were performed in accordance with the biosafety guidelines of the National Institute of Infectious Diseases and relevant national regulations for handling BSL-2 organisms.

### Phage isolation

Phages were isolated from purified sewage collected from Tokyo, Japan. Sewage samples were first centrifuged (6,000 × g, room temperature, 15 min) to remove large debris. To the supernatant, 10% w/v polyethylene glycol 8000 and 4% w/v NaCl were added, dissolved, and incubated overnight at 4 ℃. The following day, samples were centrifuged (8,000 × g, 4 ℃, 60 min) and the pellet was resuspended in 1 mL SM buffer (10mM MgSO_4_, 100mM NaCl, 0.01% w/v gelatin, and 50mM Tris-HCl [pH7.5]). The suspended pellet was purified by 0.45 μm filtration and the stored at 4 ℃. To isolate phages, 10 µL of concentrated sewage and 100 µL overnight culture were mixed to 3 mL of molten soft agar (1 mM CaCl_2_) and poured to LB agar plates. Plaques were picked to SM buffer, serially diluted, and spotted on their respective host to obtain pure phage. To propagate, single plaques were picked to 100 µL SM buffer and added to 2 mL LB (10 g/L polypeptone, 10 g/L sodium chloride and 5 g/L yeast extract) with 10 µL overnight culture. After around 2 h, lysates were purified by 0.45 μm filtration.

### Host range spot assay

Phage host range was determined by spot assay. Purified phage was 10-fold serially diluted and 2 µL was plated to double layer agar plates. Double layer agar plates were made by mixing 100 µL overnight culture to 3 mL molten agar (1 mM CaCl_2_) and overlaying the mixture to agar plates. Efficiency of plating (EOP) was calculated by comparing the phage titer (PFU/mL) plated on the strain of interest to the phage titer (PFU/mL) plated on the isolation strain.

### Phage genome extraction

Phage DNA was prepared following the previously described method^24^. Phages were propagated and purified by PEG-concentration before genome extraction. Overnight host culture was inoculated 2% to 20 mL of LB and incubated at 37 ℃ with shaking for 1–2 h. To this, 20 µL CaCl_2_ (1 M) and 20 µL phages were added (~ 10^9^ – 10^10^ PFU/mL) and further incubated at 37 ℃ with shaking until clear. Lysates were centrifuged (8,000 × *g*, 15 min, 20 ℃) and the supernatant was sterilized by 0.45 μm filtration. To the supernatants, 1.7 µL DNase I (1 U/mL) and 2 µL RNase A (10 µg/mL) were added and incubated at 37 ℃ for 1 h. To this, an equal volume of PEG-8000 solution (10% w/v PEG 8000, 1 M NaCl, 5 mM Tris-HCl [pH 7.5], 5 mM MgSO_4_) was added and incubated at 4 ℃ overnight. The following day, phages were pelleted by centrifugation (10,000 × *g*, 30 min, 4 ℃). The supernatant was decanted, and pellets were used for genome extraction using a DNeasy Blood and Tissue Kit (Qiagen, Japan) following manufacturer specifications. Once purified, genomes were sent for sequencing.

### Sequencing, genome assembly, and phylogenetic analysis

Purified phage DNA was submitted to Azenta (https://www.genewiz.com/ja-jp/public/services/next-generation-sequencing) for sequencing by Illumina Miseq (sequencing depth = 60-fold). Library preparation was done by Azenta following the standard Whole Genome Sequencing Universal protocol. In short, DNA was fragmented to an average size of 350 bp and underwent end-repair and A-tailing. Adapters were ligated and fragments were amplified before being size selected, purified, and sequenced. Contigs were trimmed using Trimmomatic v0.39^25^, assembled using Unicycler v0.5.1^26^, and annotated by Pharokka v1.7.4^27^. All packages were run using default parameters. Assembled genomes were compared to the BLASTn database, with the hits (Query coverage ≥ 80%, Percent Identity ≥ 80%) of each being used to construct a proteomic tree using ViPTree (https://www.genome.jp/viptree/)^28^. The top BLAST hits were used to construct each tree. Additionally, the three phages were included in a reference tree using representative samples (https://www.genome.jp/virushostdb/). The average nucleotide identity (ANI) was used to determine the novelty of the isolated phages(https://www.ezbiocloud.net/tools/ani)^29^. Genomes with an ANI > 95% were considered the same species per the International Committee on Taxonomy of Viruses (ICTV) guidelines^29^. VIRDIC was also used to determine the intergenomic distances between samples and was accessed via web-service (https://rhea.icbm.uni-oldenburg.de/viridic/)^30^.

### Transmission electron microscopy

Phage samples were purified by CsCl density gradient ultracentrifugation for TEM imaging. First, phage lysate (20 mL) was purified by 0.45 μm filtration and washed with SM buffer to a final volume of 1 mL by Amicon Ultra centrifugation (100 kDa MWCO, Merck Millipore). Samples were then subjected to ultracentrifugation (100,000 × g, 4 ℃, 1 h) and washed three times with SM buffer by Amicon Ultra centrifugation (100 kDa MWCO, Merck Millipore) to a final volume of 2 mL SM buffer. For TEM imaging, formvar and carbon coated copper mesh grids (Veco grids, Nisshin EM, Tokyo, Japan) were glow-discharged. To this, samples were dropped and set for 1 min. Grids were then washed and stained with 2% uranium acetate, and imaged (HT7700, Hitachi Ltd., Japan) at 80 kV.

### One-step growth

Phage latent period and burst size were calculated from one-step growth curves. Overnight culture was diluted 50-fold and incubated at 37 ℃ with shaking until the OD600 reached 0.2. After reaching 0.2, phage was added to a multiplicity of infection (MOI) of 0.1 and incubated at 37 ℃ for 5 min with shaking. Cultures were then pelleted gently (4,000 × *g*, 2 min, 20 ℃), washed once with LB, and resuspended in 10 mL prewarmed LB and immediately sampled. From this point, samples were taken every 5 min for 40 min. The samples were immediately 10-fold serially diluted and spotted to the respective host. The latent period was found by determining the time in which the phage titer reached the initial phage titer. The burst size was found by dividing the difference of the final phage titer and initial phage titer by the initial titer.

## Supplementary Information

Below is the link to the electronic supplementary material.

Supplementary Material 1

## Data Availability

Genome sequences of Enterobacter cloacae complex phages vB_ECC_CW742, vB_ECC_MY742, and vB_ECC_YI837 were registered to GenBank under GenBank accession PV019367, PV593737, and PV593738, respectively.
