# Supplementary material for: Isolation and characterization of bacteriophages for carbapenem resistant Enterobacter cloacae complex strains
Source: Sci Rep. 2025 Nov 6;15:38914. doi: 10.1038/s41598-025-22703-x (PMC12592470; doi:10.1038/s41598-025-22703-x)
Supplement: Supplementary file 1 — Supplementary Material 1 [file 41598_2025_22703_MOESM1_ESM.docx]

**Supplementary Table 1** ECC clinical isolation information.

| ST | Library Number | ID | BioSample accession no. | Location |
| --- | --- | --- | --- | --- |
| 13 | 399 | JBCBAAD-19-0081 | SAMD00499530 | Japan:Gifu |
| 19 | 365 | JBBDABG-19-0032 | SAMD00498692 | Japan:Tokyo |
| 20 | 6 | JBAGABE-19-0080 | SAMD00497831 | Japan:Yamagata |
| 20 | 435 | JBAGABE-19-0109 | SAMD00497837 | Japan:Yamagata |
| 24 | 85 | JBBEABA-19-0004 | SAMD00499174 | Japan:Kanagawa |
| 24 | 351 | JBBDAAF-19-0005 | SAMD00498494 | Japan:Tokyo |
| 27 | 94 | JBDEACE-19-0079 | SAMD00501502 | Japan:Hiroshima |
| 32 | 84 | JBBEAAJ-19-0007 | SAMD00499162 | Japan:Kanagawa |
| 32 | 88 | JBBEABG-19-0001 | SAMD00499185 | Japan:Kanagawa |
| 32 | 182 | JBBEAAJ-19-0011 | SAMD00499164 | Japan:Kanagawa |
| 41 | 442 | JBEAACH-19-0004 | SAMD00502033 | Japan:Fukuoka |
| 50 | 408 | JBAIAAD-19-0115 | SAMD00497889 | Japan:Ibaraki |
| 51 | 172 | JBEHAAB-19-0043 | SAMD00502433 | Japan:Okinawa |
| 57 | 491 | JBBDAGI-19-0039 | SAMD00499058 | Japan:Tokyo |
| 62 | 166 | JBEAAAD-19-0096 | SAMD00501940 | Japan:Fukuoka |
| 62 | 172 | JBEAACG-19-0081 | SAMD00502026 | Japan:Fukuoka |
| 62 | 176 | JBEAAEE-19-0001 | SAMD00502074 | Japan:Fukuoka |
| 62 | 181 | JBEAAEE-19-0050 | SAMD00502090 | Japan:Fukuoka |
| 62 | 186 | JBEAAEE-19-0140 | SAMD00502103 | Japan:Fukuoka |
| 62 | 198 | JBEBAAB-19-0102 | SAMD00502301 | Japan:Saga |
| 62 | 448 | JBEAACG-19-0092 | SAMD00502028 | Japan:Fukuoka |
| 66 | 29 | JBBDAAB-19-0011 | SAMD00498458 | Japan:Tokyo |
| 68 | 440 | JBEAACH-19-0001 | SAMD00502030 | Japan:Fukuoka |
| 78 | 28 | JBBDAAB-19-0007 | SAMD00498455 | Japan:Tokyo |
| 78 | 38 | JBBDAAF-19-0040 | SAMD00498522 | Japan:Tokyo |
| 78 | 70 | JBBDAFI-19-0029 | SAMD00498962 | Japan:Tokyo |
| 78 | 75 | JBBDAJA-19-0064 | SAMD00499090 | Japan:Tokyo |
| 78 | 140 | JBCIAAB-19-0172 | SAMD00500663 | Japan:Hyogo |
| 78 | 163 | JBDJAAE-19-0069 | SAMD00501842 | Japan:Kochi |
| 78 | 177 | JBEAAEE-19-0002 | SAMD00502075 | Japan:Fukuoka |
| 78 | 188 | JBEAAEE-19-0155 | SAMD00502107 | Japan:Fukuoka |
| 78 | 191 | JBEAAFA-19-0086 | SAMD00502170 | Japan:Fukuoka |
| 78 | 497 | JBBDAGI-19-0100 | SAMD00499075 | Japan:Tokyo |
| 90 | 23 | JBBCAAG-19-0002 | SAMD00498274 | Japan:Chiba |
| 90 | 83 | JBBEAAJ-19-0005 | SAMD00499161 | Japan:Kanagawa |
| 90 | 181 | JBBEAAJ-19-0008 | SAMD00499163 | Japan:Kanagawa |
| 93 | 247 | JBCHAFC-19-0064 | SAMD00500621 | Japan:Osaka |
| 99 | 432 | JBAGABE-19-0062 | SAMD00497829 | Japan:Yamagata |
| 108 | 409 | JBCIACA-19-0636 | SAMD00500850 | Japan:Hyogo |
| 113 | 101 | JBDJAAE-19-0102 | SAMD00501854 | Japan:Kochi |
| 114 | 243 | JBCHAFC-19-0027 | SAMD00500610 | Japan:Osaka |
| 116 | 41 | JBBDAAJ-19-0051 | SAMD00498602 | Japan:Tokyo |
| 125 | 265 | JBBCACA-19-0104 | SAMD00498328 | Japan:Chiba |
| 127 | 86 | JBBEABA-19-0021 | SAMD00499179 | Japan:Kanagawa |
| 133 | 18 | JBBBAAB-19-0138 | SAMD00498101 | Japan:Saitama |
| 133 | 30 | JBBDAAB-19-0067 | SAMD00498467 | Japan:Tokyo |
| 133 | 35 | JBBDAAF-19-0018 | SAMD00498507 | Japan:Tokyo |
| 133 | 37 | JBBDAAF-19-0029 | SAMD00498514 | Japan:Tokyo |
| 133 | 50 | JBBDABE-19-0091 | SAMD00498681 | Japan:Tokyo |
| 133 | 57 | JBBDADH-19-0027 | SAMD00498789 | Japan:Tokyo |
| 133 | 73 | JBBDAGF-19-0034 | SAMD00499027 | Japan:Tokyo |
| 133 | 76 | JBBDAJB-19-0014 | SAMD00499141 | Japan:Tokyo |
| 133 | 77 | JBBDAJB-19-0015 | SAMD00499142 | Japan:Tokyo |
| 133 | 87 | JBBEABA-19-0025 | SAMD00499180 | Japan:Kanagawa |
| 133 | 96 | JBBEABG-19-0028 | SAMD00499202 | Japan:Kanagawa |
| 133 | 165 | JBEAAAD-19-0047 | SAMD00501928 | Japan:Fukuoka |
| 133 | 179 | JBEAAEE-19-0042 | SAMD00502087 | Japan:Fukuoka |
| 133 | 371 | JBBDAGF-19-0069 | SAMD00499037 | Japan:Tokyo |
| 151 | 528 | JBABADI-19-0083 | SAMD00497511 | Japan:Hokkaido |
| 171 | 133 | JBCHAEJ-19-0037 | SAMD00500569 | Japan:Osaka |
| 175 | 15 | JBAJAAJ-19-0001 | SAMD00497979 | Japan:Tochigi |
| 175 | 45 | JBBDABA-19-0004 | SAMD00498614 | Japan:Tokyo |
| 175 | 180 | JBEAAEE-19-0043 | SAMD00502088 | Japan:Fukuoka |
| 175 | 106 | JBBDAFG-19-0124 | SAMD00498877 | Japan:Tokyo |
| 175 | 478 | JBCDABH-19-0013 | SAMD00499884 | Japan:Aichi |
| 182 | 531 | JBAFAAB-19-0016 | SAMD00497749 | Japan:Akita |
| 189 | 18 | JBCIAEJ-19-0009 | SAMD00500937 | Japan:Hyogo |
| 190 | 21 | JBBBAEF-19-0073 | SAMD00498247 | Japan:Saitama |
| 190 | 453 | JBCBAAD-19-0109 | SAMD00499559 | Japan:Gifu |
| 204 | 68 | JBBDAFH-19-0097 | SAMD00498944 | Japan:Tokyo |
| 204 | 199 | JBEDACG-19-0107 | SAMD00502329 | Japan:Kumamoto |
| 204 | 420 | JBEDACG-19-0108 | SAMD00502330 | Japan:Kumamoto |
| 252 | 13 | JBAIAAD-19-0002 | SAMD00497877 | Japan:Ibaraki |
| 252 | 14 | JBAIAAD-19-0071 | SAMD00497869 | Japan:Ibaraki |
| 252 | 20 | JBBBAAI-19-0002 | SAMD00498133 | Japan:Saitama |
| 252 | 123 | JBCEACC-19-0030 | SAMD00500102 | Japan:Mie |
| 252 | 434 | JBAGABE-19-0101 | SAMD00497836 | Japan:Yamagata |
| 258 | 487 | JBEAAFA-19-0587 | SAMD00502262 | Japan:Fukuoka |
| 269 | 406 | JBEAAEI-19-0188 | SAMD00502149 | Japan:Fukuoka |
| 279 | 117 | JBEHABI-19-0188 | SAMD00502461 | Japan:Okinawa |
| 280 | 451 | JBCBAAD-19-0101 | SAMD00499556 | Japan:Gifu |
| 304 | 397 | JBEHAAB-19-0192 | SAMD00502407 | Japan:Okinawa |
| 373 | 1 | JBABADI-19-0057 | SAMD00497507 | Japan:Hokkaido |
| 414 | 32 | JBEHABI-19-0050 | SAMD00502503 | Japan:Okinawa |
| 480 | 446 | JBEAACH-19-0012 | SAMD00502041 | Japan:Fukuoka |
| 484 | 44 | JBBDABA-19-0003 | SAMD00498613 | Japan:Tokyo |
| 484 | 46 | JBBDABA-19-0005 | SAMD00498615 | Japan:Tokyo |
| 484 | 53 | JBBDACG-19-0013 | SAMD00498719 | Japan:Tokyo |
| 484 | 108 | JBCCAAB-19-0087 | SAMD00499629 | Japan:Shizuoka |
| 484 | 131 | JBCHACG-19-0448 | SAMD00500488 | Japan:Osaka |
| 484 | 296 | JBBDABA-19-0006 | SAMD00498616 | Japan:Tokyo |
| 486 | 433 | JBAGABE-19-0082 | SAMD00497833 | Japan:Yamagata |
| 501 | 229 | JBCHACG-19-0259 | SAMD00500449 | Japan:Osaka |
| 524 | 329 | JBDGAAB-19-0038 | SAMD00501581 | Japan:Tokushima |
| 526 | 314 | JBABADF-19-0129 | SAMD00497424 | Japan:Hokkaido |
| 539 | 19 | JBCIAEJ-19-0013 | SAMD00500939 | Japan:Hyogo |
| 544 | 483 | JBDCAAE-19-0492 | unregistered | Japan:Shimane |
| 563 | 105 | JBCAAAI-19-0110 | SAMD00499479 | Japan:Nagano |
| 636 | 280 | JBBGAAF-19-0022 | SAMD00499385 | Japan:Toyama |
| 666 | 193 | JBEAAAI-19-0041 | SAMD00501967 | Japan:Fukuoka |
| 691 | 362 | JBDEABG-19-0193 | SAMD00501428 | Japan:Hiroshima |
| 702 | 104 | JBCHAFC-19-0014 | SAMD00500596 | Japan:Osaka |
| 705 | 22 | JBDDAAF-19-0084 | SAMD00501241 | Japan:Okayama |
| 742 | 8 | JBAGABE-19-0098 | SAMD00497835 | Japan:Yamagata |
| 742 | 11 | JBAGABE-19-0120 | SAMD00497844 | Japan:Yamagata |
| 742 | 24 | JBBCAAG-19-0007 | SAMD00498279 | Japan:Chiba |
| 742 | 26 | JBBCADC-19-0142 | SAMD00498386 | Japan:Chiba |
| 742 | 27 | JBBCAEG-19-0003 | SAMD00498403 | Japan:Chiba |
| 742 | 31 | JBBDAAB-19-0170 | SAMD00498436 | Japan:Tokyo |
| 742 | 42 | JBBDAAJ-19-0055 | SAMD00498603 | Japan:Tokyo |
| 742 | 454 | JBBCADC-19-0145 | SAMD00498387 | Japan:Chiba |
| 769 | 390 | JBCBAAJ-19-0100 | SAMD00499614 | Japan:Gifu |
| 807 | 429 | JBCGACB-19-0312 | SAMD00500278 | Japan:Kyoto |
| 837 | 101 | JBBEADF-19-0334 | SAMD00499306 | Japan:Kanagawa |
| 837 | 486 | JBEAAFA-19-0567 | SAMD00502259 | Japan:Fukuoka |
| 893 | 468 | JBBDAGD-19-0254 | SAMD00499004 | Japan:Tokyo |
| 963 | 180 | JBEFAAC-19-0045 | SAMD00502361 | Japan:Miyazaki |
| 982 | 327 | JBDGAAB-19-0032 | SAMD00501577 | Japan:Tokushima |
| 1017 | 457 | JBDGAAB-19-0117 | SAMD00501635 | Japan:Tokushima |
| 1051 | 23 | JBDDAAF-19-0087 | SAMD00501243 | Japan:Okayama |
| 1057 | 499 | JBBDADH-19-0034 | SAMD00498790 | Japan:Tokyo |
| 1288 | 458 | JBDGAAB-19-0118 | SAMD00501636 | Japan:Tokushima |
| 1348 | 307 | JBCHACG-19-0389 | SAMD00500415 | Japan:Osaka |
| 1352 | 56 | JBDIABB-19-0090 | SAMD00501784 | Japan:Ehime |
| 1399 | 370 | JBBDAGF-19-0064 | SAMD00499035 | Japan:Tokyo |
| 1578 | 542 | JBAFAAB-19-0054 | SAMD00497767 | Japan:Akita |
| 1659 | 228 | JBCHACG-19-0258 | SAMD00500448 | Japan:Osaka |
| 1681 | 394 | JBEHAAB-19-0123 | SAMD00502396 | Japan:Okinawa |
| 1780 | 391 | JBCDACH-19-0001 | SAMD00499885 | Japan:Aichi |
| 1939 | 256 | JBABAHA-19-0025 | SAMD00497612 | Japan:Hokkaido |


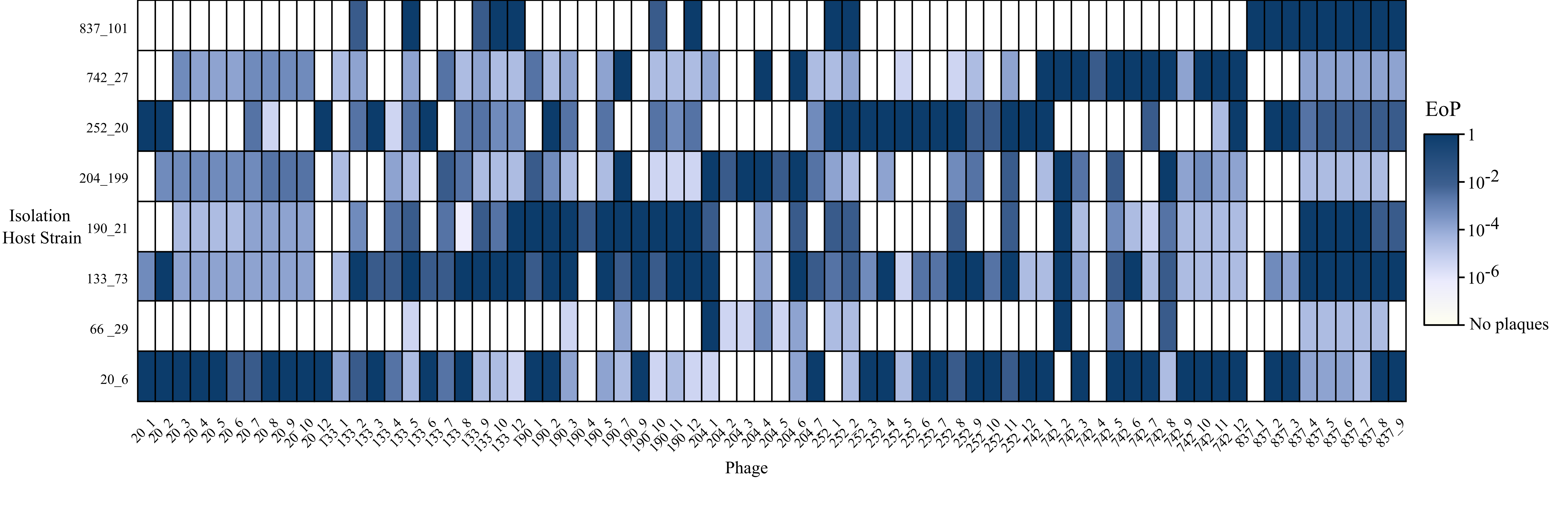
**Supplementary Figure 1.** Initial host range of phage isolates against screening hosts. The numbers on the Y-axis indicate the host ST followed by the catalog entry within our library (13_399: ST 13, #399). The numbers on X-axis indicate the phage isolation host and replicate number (20_1: isolated from ST20, replicate 1). Phages that produced no plaques against all of the isolation host strains were not included. Φ742_1, Φ742_2, and Φ837_9 were selected for further study. Φ837_9 was referred to as Φ837 in the main text.


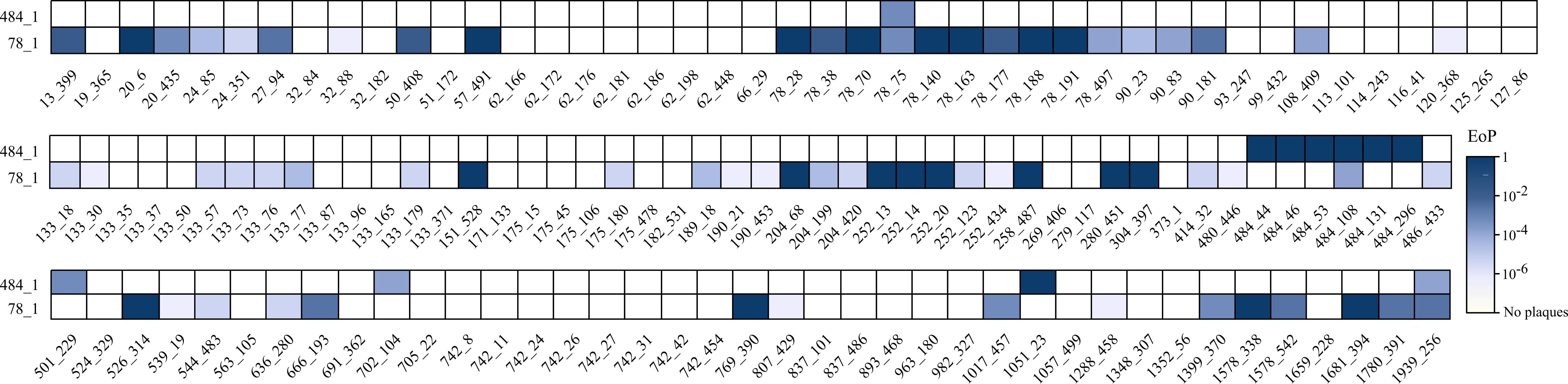


**Supplementary Figure 2.** Host range of Φ78_1 and Φ484_1. The numbers below each tile indicate the host ST followed by the catalog entry within our library (13_399: ST 13, #399). EOP was determined by comparing the PFU/mL of the selected strain against the PFU/mL of the isolation host.

| **Phage** | **vB_ECC_CW742** |
| --- | --- |
| vB_ECC_CW742 | 100 |
| PQ140450.1 | 87.978 |
| PP597396.1 | 86.649 |
| PQ932598.1 | 86.239 |
| MN013084.1 | 86.18 |
| NC_055726.1 | 85.822 |
| ON585039.1 | 85.786 |
| NC_014662.1 | 85.743 |
| OP558005.1 | 85.579 |
| OL828290.1 | 85.568 |
| OQ721913.1 | 85.438 |
| PQ096439.1 | 85.335 |
| PQ824230.1 | 85.013 |
| ON212265.1 | 84.942 |
| OQ721912.1 | 84.936 |
| NC_023561.1 | 84.851 |
| PP212877.1 | 84.771 |
| ON454249.1 | 84.553 |
| ON212267.1 | 84.484 |
| OR567511.1 | 84.063 |
| NC_055739.1 | 83.995 |
| OR753410.1 | 83.899 |
| OL849997.1 | 83.872 |
| PQ096440.1 | 83.361 |
| OL828291.1 | 82.606 |
| PQ096442.1 | 82.581 |
| PQ898399.1 | 69.688 |

**Supplementary Table 2** vB_ECC_CW742 VIRDIC similarity scores.

| **Phage** | **vB_ECC_CW742** |
| --- | --- |
| vB_ECC_CW742 | 100 |
| vB_ECC_MY742 | 38.218 |

| **Phage** | **vB_ECC_MY742** |
| --- | --- |
| vB_ECC_MY742 | 100 |
| NC_048849.1 | 91.157 |
| ON630910.1 | 90.823 |
| OL355124.1 | 90.024 |
| NC_070777.1 | 86.371 |
| NC_070778.1 | 86.195 |
| NC_070776.1 | 85.574 |
| PP236086.1 | 85.046 |

**Supplementary Table 3** vB_ECC_MY742 VIRDIC similarity scores.

**Supplementary Table 4** vB_ECC_YI837VIRDIC similarity scores.

| **Phage** | **vB_ECC_YI837** |
| --- | --- |
| vB_ECC_YI837 | 100 |
| PV417214.1 | 96.235 |
| KX431560.1 | 95.815 |
| PP337149.1 | 95.332 |
| NC_048645.1 | 95.041 |
| LC589952.1 | 94.957 |
| ON157416.1 | 94.852 |
| PP889466.1 | 94.459 |
| NC_028755.1 | 94.448 |
| PP889464.1 | 94.236 |
| PQ096441.1 | 94.071 |
| MH823906.1 | 93.955 |
| MT341500.1 | 93.899 |
| MN508621.2 | 93.834 |
| MW629017.1 | 93.552 |
| PQ096438.1 | 93.538 |
| OP970827.1 | 93.317 |
| PV067695.1 | 93.062 |
| OL539470.1 | 92.941 |
| NC_048646.1 | 92.096 |
| MW021756.1 | 91.785 |
| PP554394.1 | 91.646 |
| OL539471.1 | 91.43 |
| MW021751.1 | 91.391 |
| OR637326.1 | 90.778 |
| MN508624.2 | 90.528 |
| PP889458.1 | 90.517 |
| PP738789.1 | 90.494 |
| MN508623.2 | 90.446 |
| PV425437.1 | 90.249 |
| MW767161.1 | 90.008 |
| OR637327.1 | 89.904 |
| OQ571799.1 | 89.477 |
| NC_019398.1 | 89.331 |
| MW250785.1 | 88.432 |
| MN508622.2 | 87.918 |
| OL355131.1 | 87.499 |
| OL355127.1 | 87.087 |
| OL355125.1 | 87.072 |
| OL355128.1 | 87.063 |
| OL551674.1 | 86.841 |
| OL355129.1 | 86.019 |
| NC_014467.1 | 76.915 |
| NC_025414.1 | 76.669 |
| NC_007023.1 | 76.437 |
| HE858210.2 | 76.43 |
| NC_029013.1 | 76.195 |
| MT334653.1 | 76.178 |
| NC_021344.2 | 76.145 |
| HE981739.1 | 76.126 |
| MW021749.1 | 75.811 |
| NC_031057.1 | 75.526 |

**Supplementary Table 5** Pharokka genome annotation summary.

|  | Number of hits | | |
| --- | --- | --- | --- |
|  | Φ742_1/ vB_ECC_CW742 | Φ742_2/ vB_ECC_MY742 | Φ837/ vB_ECC_YI837 |
| ORFs | 309 | 313 | 286 |
| Connector | 3 | 3 | 3 |
| DNA, RNA and nucleotide metabolism | 46 | 49 | 42 |
| Head and packaging | 17 | 17 | 13 |
| Integration and excision | 0 | 0 | 0 |
| Lysis | 7 | 7 | 7 |
| Moron, auxiliary metabolic gene, and host takeover | 7 | 11 | 4 |
| Other | 13 | 12 | 14 |
| Tail | 29 | 29 | 30 |
| Transcription regulation | 8 | 8 | 5 |
| Unknown function | 179 | 177 | 168 |
| tRNAs | 19 | 9 | 1 |
| CRISPRs | 0 | 0 | 0 |
| tmRNAs | 0 | 0 | 0 |
| VFDB_Virulence_Factors | 0 | 0 | 0 |
| CARD_AMR_Genes | 0 | 0 | 0 |

**
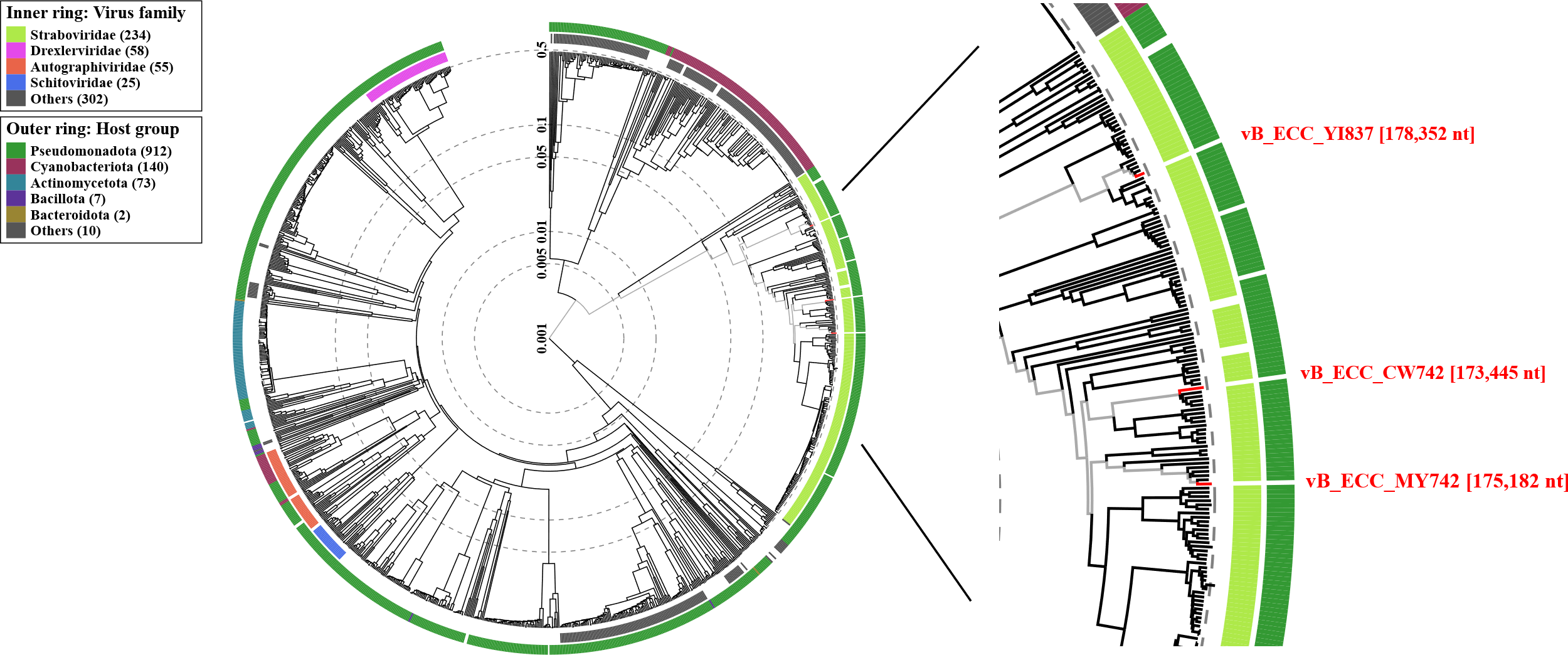
**

**Supplementary Figure 3** VIRAL proteomic tree of reference viruses and three phages isolated in this study. Reference virus data can be found at Virus-Host DB (https://www.genome.jp/virushostdb/).

**
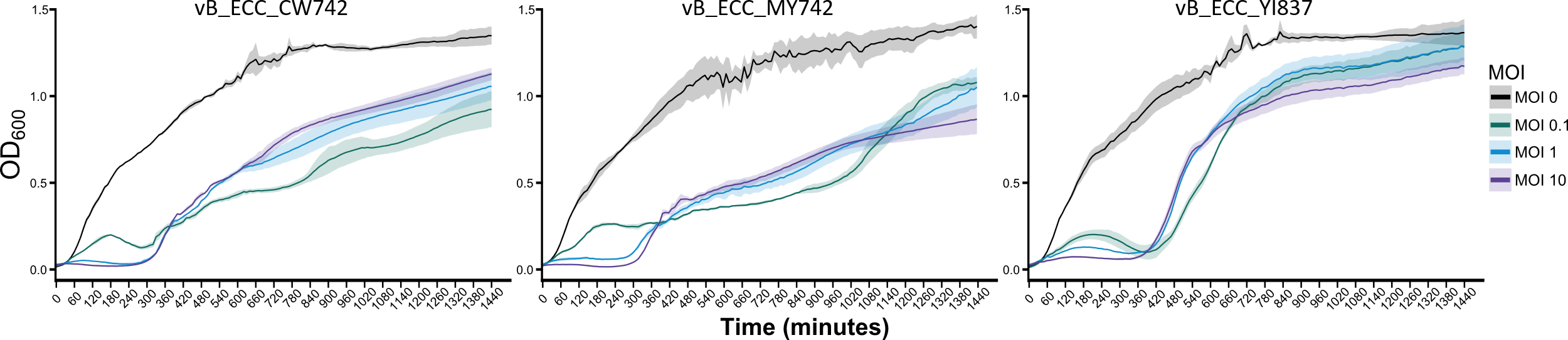
**

**Supplementary Figure 4** Growth kinetics of the three isolated phages. Bacteria were infected at T=0 and the OD600 was measured every 10 minutes for 24 hours. The average of three replicates was plotted, with the shaded region representing the standard deviation.
